# Molecular analysis of the *PAX6* gene in Mexican patients with congenital aniridia: report of four novel mutations

**Published:** 2008-09-08

**Authors:** Camilo E. Villarroel, Cristina Villanueva-Mendoza, Lorena Orozco, Miguel Angel Alcántara-Ortigoza, Diana F. Jiménez, Juan C. Ordaz, Ariadna González-del Angel

**Affiliations:** 1 Department of Human Genetics, National Institute of Pediatrics, Mexico City, Mexico; 2 Department of Ophthalmology, National Institute of Pediatrics, Mexico City, Mexico; 3 Department of Genetics, Hospital Dr. Luis Sanchez Bulnes, Asociación Para Evitar la Ceguera en México, Mexico City, Mexico; 4 Department of Ophthalmology, Hospital Dr. Luis Sánchez Bulnes, Asociación Para Evitar la Ceguera en México, Mexico City, Mexico; 5 The National Institute of Genomic Medicine, Mexico City, Mexico

## Abstract

**Purpose:**

Paired box gene 6 (*PAX6)* heterozygous mutations are well known to cause congenital non-syndromic aniridia. These mutations produce primarily protein truncations and have been identified in approximately 40%–80% of all aniridia cases worldwide. In Mexico, there is only one previous report describing three intragenic deletions in five cases. In this study, we further analyze *PAX6* variants in a group of Mexican aniridia patients and describe associated ocular findings.

**Methods:**

We evaluated 30 nonrelated probands from two referral hospitals. Mutations were detected by single-strand conformation polymorphism (SSCP) and direct sequencing, and novel missense mutations and intronic changes were analyzed by in silico analysis. One intronic variation (IVS2+9G>A), which in silico analysis suggested had no pathological effects, was searched in 103 unaffected controls.

**Results:**

Almost all cases exhibited phenotypes that were at the severe end of the aniridia spectrum with associated ocular alterations such as nystagmus, macular hypoplasia, and congenital cataracts. The mutation detection rate was 30%. Eight different mutations were identified: four (c.184_188dupGAGAC, c.361T>C, c.879dupC, and c.277G>A) were novel, and four (c.969C>T, IVS6+1G>C, c.853delC, and IVS7–2A>G) have been previously reported. The substitution at position 969 was observed in two patients. None of the intragenic deletions previously reported in Mexican patients were found. Most of the mutations detected predict either truncation of the PAX6 protein or conservative amino acid changes in the paired domain. We also detected two intronic non-pathogenic variations, IVS9–12C>T and IVS2+9G>A, that had been previously reported. Because the latter variation was considered potentially pathogenic, it was analyzed in 103 healthy Mexican newborns where we found an allelic frequency of 0.1116 for the A allele.

**Conclusions:**

This study adds four novel mutations to the worldwide *PAX6* mutational spectrum, and reaffirms the finding that c.969C>T is one of the three more frequent causal mutations in aniridia cases. It also provides evidence that IVS2+9G>A is an intronic change without pathogenic effect.

## Introduction

Aniridia is a congenital ocular disorder characterized by bilateral variable iris hypoplasia with an estimated occurrence of one in every 64,000–96,000 live births worldwide [1]. The manifestations of the aniridia phenotype are variable, ranging from thinning of the stroma and absent pupillary sphincter to complete aniridia [2,3]. In addition to iris hypoplasia, other ocular congenital defects may be present such as cataracts, foveal hypoplasia, nystagmus, corneal opacity, lens dislocation, and glaucoma with significant loss of vision [4]. Because of the wide spectrum of clinical manifestations associated with this ocular pathology, Gronskov et al. [5] proposed to categorize the phenotype into six different levels based on iris presentation. However, this classification is not widely used.

Approximately two thirds of cases are familial with an autosomal dominant inheritance pattern, probably with complete penetrance [5,6]. Some sporadic aniridia cases have the WAGR syndrome (Wilms tumor, aniridia, genitourinary anomalies, and mental retardation; OMIM 194072). Several genes at 11p13 are deleted in the WAGR syndrome including *WT1* and the evolutionarily conserved paired box gene 6 (*PAX6*) [7].

The human *PAX6* spans 26 kilobases (kb), contains 14 exons [8,9], and encodes the PAX6 transcription factor. *PAX6* is considered the master control gene for ocular morphogenesis and contributes to central nervous system development [10]. Like other transcriptional activators of the PAX family, PAX6 contains two DNA-binding domains (a paired domain at the NH_2_-terminus and a middle homeodomain) and a proline-serine-threonine (PST)-rich transactivator domain at the COOH-terminus [8,9].

Homozygous loss of *PAX6* is thought to lead to early embryonic lethality [11]. Heterozygous mutations are found in approximately 40%–80% of all non-syndromic aniridia cases [9,12-15],  and most are searched by single strand conformation polymorphism (SSCP), which is considered one of the most useful molecular detection methods [12,16]. There are no clear gene hotspots, and the majority of mutations in *PAX6* are predicted to introduce premature termination codons, most of which are assumed to be functionally null because of haploinsufficiency [15]. To date, more than 400 *PAX6* mutations have been reported (Online Human PAX6 Allelic Database). The most frequent mutations are c.1080C>T (c.718C>T), c.969C>T (c. 607C>T), c.1311C>T (c.949C>T), and c.1629insT (c.1267dupT).

The molecular basis of aniridia in Mexico is poorly characterized. In fact, there is only one report of three different intragenic deletions of *PAX6* found in five unrelated cases in the Mexican population. Interestingly, the authors of this study suggested a founder effect for a four-base intragenic deletion (c.732_735delAACA) in exon 7 in Mexican aniridia patients because this mutation was found in three nonrelated cases [17]. In the present study, we further analyze *PAX6* variants in a group of Mexican aniridia patients and describe associated ocular findings.

## Methods

We evaluated 30 unrelated aniridia probands recruited from two referral hospitals in Mexico City, the National Institute of Pediatrics and the Dr. Luis Sanchez Bulnes Hospital. All individuals were of Mexican origin, showed no associated systemic abnormalities, and had normal psychomotor development. Patients were categorized according to Gronskov’s iris classification [5].

This study was conducted in accordance with the World Medical Association Declaration of Helsinki and was approved by the respective local research and ethics committees. Written informed consent was obtained from all participants.

Genomic DNA was extracted from peripheral blood leukocytes using the PureGene DNA purification kit (Gentra Systems, Minneapolis, MN). *PAX6* mutation screening was performed by polymerase chain reaction (PCR) amplification of all 14 exons and immediate flanking sequences using the primers and conditions proposed by Love et al. [18] followed by SSCP analysis in 1X Mutation Detection Enhancement gels (BioWhittaker Molecular Applications, Rockland, ME). Gels were run under constant power (6 W) for 12 h at room temperature and visualized by silver nitrate staining (Silver Stain Kit, Bio-Rad Laboratories, Hercules, CA). Fragments displaying abnormal electrophoretic patterns were purified by the silica column method (QIAquick, Gel Extraction Kit; QIAGEN Inc. Valencia CA) and directly sequenced using a Big Dye Terminator Kit with an automated ABI PRISM Model 377 sequencer (Applied Biosystems, Foster City, CA) according to the manufacturer’s recommendations. The mutations identified in the probands were sought in parents that were available. The nomenclature used for describing novel genetic changes follows the recommendations of the Human Genome Variation Society [19], and nucleotides were numbered according to the consensus coding DNA sequence of *PAX6* isoform a (CCDS31451.1). In silico analyses of novel missense mutations and intronic changes were performed using the SIFT program and the NetGene2 Server, respectively. The intronic nucleotide variation, IVS2+9G>A (c.-129+9G>A), reported previously as pathogenic [20], was sought in 103 nonrelated healthy Mexican newborns using the PCR restriction fragment length polymorphism (PCR-RFLP) method by amplifying the 3′ end of exon 2 according to Love et al. [18] and restricting with the AciI enzyme where the presence of the G allele eliminates the restriction site. The Hardy–Weinberg equilibrium conformance was evaluated using the SNPstats software.

## Results

Phenotypic information was available from 28 of the 30 probands, and a summary of findings is given in Table 1. The median age of cases was 5.2 years, and 18 of the probands (62%) were female. Eighteen of the cases (62%) were sporadic cases, and 11 had at least one relative with aniridia. Absent or nearly absent irides were evident in 26 cases (93%), and these were categorized as Iris 5 or Iris 6 according to Gronskov’s classification [5]. Of the remaining two cases, one was classified as Iris 3 and 4 (one eye each) and the other was classified as Iris 4. At least two ocular-associated alterations were present in 21 patients (75%), and the most common alterations were nystagmus (75%), macular hypoplasia (57%), and congenital cataracts (53%). Other less frequent features were optic nerve hypoplasia and keratopathy. Six individuals had glaucoma, which was congenital in two cases. The iris defect was not associated with any other ocular abnormality in only one patient (case 13).

**Table 1:** Iris grade and ocular associated findings in 30 Mexican nonrelated aniridia cases.

**Case**	**Sex**	**Age (years)**	**Inheritance**	**Iris grade**	**Best corrected visual acuity**	**Nystagmus**	**Cataract**	**Glaucoma/** **treatment**	**Macular hypoplasia**	**Other**
1	F	4	Sporadic	Iris 5	20/600	+	-	-	-	-
2	M	18	Familial	Iris 4	20/200	+	+	-	+	Ptosis
3	F	11	Sporadic	Iris 6	20/100	-	+	-	-	Ptosis
4	M	0.5	Sporadic	Iris 5	FF	+	-	-	+	-
5	F	3	Sporadic	Iris 5	20/380	+	-	-	+	Ptosis, strabismus
6	F	15	Familial	Iris 3 and 4	20/25	-	+	-	-	-
7	M	33	Familial	Iris 5	FC 0.5 mt	+	+	+, SG, MD	+	Kerathopathy
8	F	3	Sporadic	Iris 6	FF	+	-	-	+	-
9	F	9	Sporadic	Iris 5	20/200	+	+	-	+	Ptosis
10	F	6	Sporadic	Iris 5	20/40	+	-	-	+	-
11	M	10	Familial	Iris 6	FC 4 mt	+	+	+, SG, MD	+	Ectopia lentis, ONH
12	M	8	Sporadic	Iris 5	FC 1 mt	+	-	congenital, SG, MD	+	Corneal leucoma
13	F	0.8	Sporadic	Iris 5	FF	-	-	-	-	-
14	M	14	Sporadic	Iris 5	20/40	-	+	-	-	-
15	F	47	Sporadic	Iris 6	FC 1.5 mt	+	+	+, SG, MD	+	Kerathopathy, ONH
16	F	0.5	Sporadic	Iris 5	FF	-	+	-	-	-
17	F	2	Sporadic	Iris 5	FF	-	-	+, MD	+	Ectopia lentis, microcornea
18	F	0.5	Sporadic	Iris 5	FF	+	-	-	+	Ectopia lentis, microcornea, ONH
19	M	16	Sporadic	Iris 6	20/200	+	+	-	-	Ptosis, strabismus
20	?	?	?	?	?	?	?	?	?	?
21	M	1	Familial	Iris 5	FF	+	-	-	+	-
22	F	8	Familial	Iris 6	20/130	+	+	-	-	-
23	M	16	Sporadic	Iris 5	20/160	+	+	-	+	-
24	M	5	Sporadic	?	?	?	?	?	?	?
25	F	13	Familial	Iris 5	20/200	+	+	-	+	Ectopia lentis
26	F	17	Sporadic	Iris 6	20/200	+	+	-	-	Strabismus
27	F	0.7	Familial	Iris 6	FF	-	+	congenital, SG, MD	?	Corneal leucoma
28	F	1	Familial	Iris 5	FF	+	-	-	+	-
29	F	6	Familial	Iris 5	20/120	+	-	-	-	-
30	M	0.4	Familial	Iris 6	FF	+	-	-	-	-

M: Male; F: Female; Iris 3: circumpupillary iris hypoplasia; Iris 4: atypical sector coloboma; Iris 5: subtotal aniridia; Iris 6: complete aniridia; FF: fix and follow; FC: finger count; +: present; -: absent; ?: information not available; SG: surgical; MD: medical; ONH: optic nerve hypoplasia.

Molecular findings are summarized in Table 2. We detected 11 SSCP mobility shifts in *PAX6* products, all of which were consistent with the presence of mutations or neutral polymorphisms after sequencing. Causal mutations of the aniridia phenotype were found in 9 of 30 cases, yielding a detection rate of 30%. All mutations were heterozygous and unique except for the recurrent mutation, c.969C>T, which was observed in two sporadic unrelated cases. Four mutations were novel, c.184_188dupGAGAC, c.361T>C, c.879dupC, and c.277G>A. The remaining four mutations identified (c.969C>T, IVS6+1G>C, c.853delC, and IVS7–2A>G) have been previously reported (Human PAX6 allelic database) . Additionally, we found two intronic, nonpathogenic variations, IVS9–12C>T and IVS2+9G>A, both of which have also been previously described [20,21]. Of the nine probands in whom pathological mutations were identified, only nine parents were available for molecular analysis (Table 2).

**Table 2:** *PAX6* gene mutations and polymorphisms identified in nine non-related Mexican aniridia cases.

**Case**	Iris grade*	Nucleotide change**	Nucleotide change***	**mRNA/** **protein effect**	**Exon/** **Domain**	**Mother´s Genotype**	**Father´s Genotype**	**Status/** **Reference**
4	Iris 5	c.184_188dupGAGAC		p.T63fsX18	Exon 6/ Paired box	Wild-type	Not available	Novel
6	Iris 3 and 4	c.361T>C		p.S121P	Exon 7/ Paired box	Heterozygous for c.361T>C	Not available	Novel
10	Iris 5	c.607C>T	c.969C>T	p.R203X	Exon 8/ Linker region	Wild-type	Not available	Previously reported (Human PAX6 allelic database)
18	Iris 5	c.357+1G>C	c.IVS6+1G>C	Cryptic donor splice-site and in-frame deletion of 36 amino acids coded by exon 6	Intron 6/ Paired box	Wild-type	Wild-type	Previously reported [22]
		Heterozygous for c.-129+9G>A	Heterozygous for IVS2+9G>A	None	Intron 2	Homozygous for G allele	Heterozygous for IVS2+9G>A (c.-129+9G>A)	Previously described as polymorphism (Human PAX6 allelic database), but also as a possible pathogenic variant [20]. Present study confirmed that it is a polymorphism
20	?	c.491delC	c.853delC	p.P164fsX43	Exon 7/ Linker region	Not available	Not available	Previously reported [5,22]
21	Iris 5	c.879dupC		p.T293fsX47	Exon 10/ PST domain	Heterozygous for c.879dupC	Wild-type	Novel
22	Iris 6	c.277G>A		p.E93K	Exon 6/ Paired box	Not available	Not available	Novel
		c.766-12C>T	IVS9-12C>T	None	Intron 9	Not available	Not available	Polymorphism previously reported [21]
24	?	c.607C>T	c.969C>T	p.R203X	Exon 8/ Linker region	Wild-type	Wild-type	Previously reported (Human PAX6 allelic database)
26	Iris 6	c.524-2A>G	IVS7-2A>G	In silico prediction: 3 cryptic acceptor splice-sites (2 out-of-frame and 1 in-frame) inside exon 8 or in-frame exon 8 skipping.	Intron 7/ Linker region	Not available	Not available	Previously reported [22]

An asterisk indicates that the measurements were according to Gronskov’s classification [5]. A question mark means that an ophthalmic evaluation was not available. A double asterisk symbol indicates that the gene mutation nomenclature was according to den Dunnen and Antonarakis [19]. A triple asterisk symbol denotes that the gene mutation nomenclature was according to previously proposed nomenclature by Ton et al. [8].

With respect to novel changes, case 4 showed an insertion of a GAGAC sequence at nucleotide position 184, causing a frameshift arising from tandem duplication of nucleotides 184–188 that is predicted to encode a protein truncated in the paired domain. At evaluation, the patient exhibited a phenotype characterized by nystagmus, macular hypoplasia, and subtotal aniridia defect (Iris 5 in Gronskov’s classification). His mother had a normal ocular phenotype and did not have the mutation. A DNA sample from his father was not available, but he was referred to as visually healthy.

**Figure 1:**
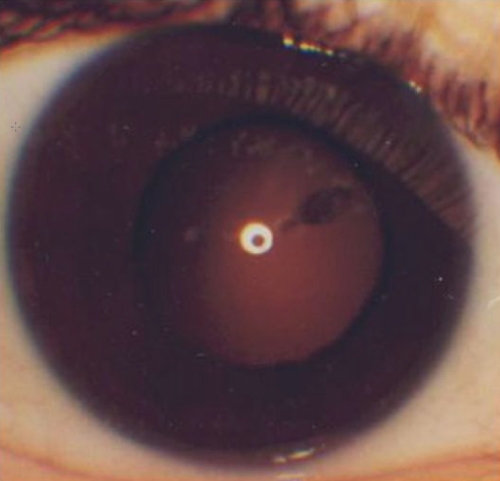
Right eye iris and pupil of aniridia case 6 who had a novel missense mutation (c.361T>C) located in the NH_2_-region of the paired domain of *PAX6*. This eye exhibited eccentric pupil and circumpupillary iris hypoplasia (Iris 3).

**Figure 2:**
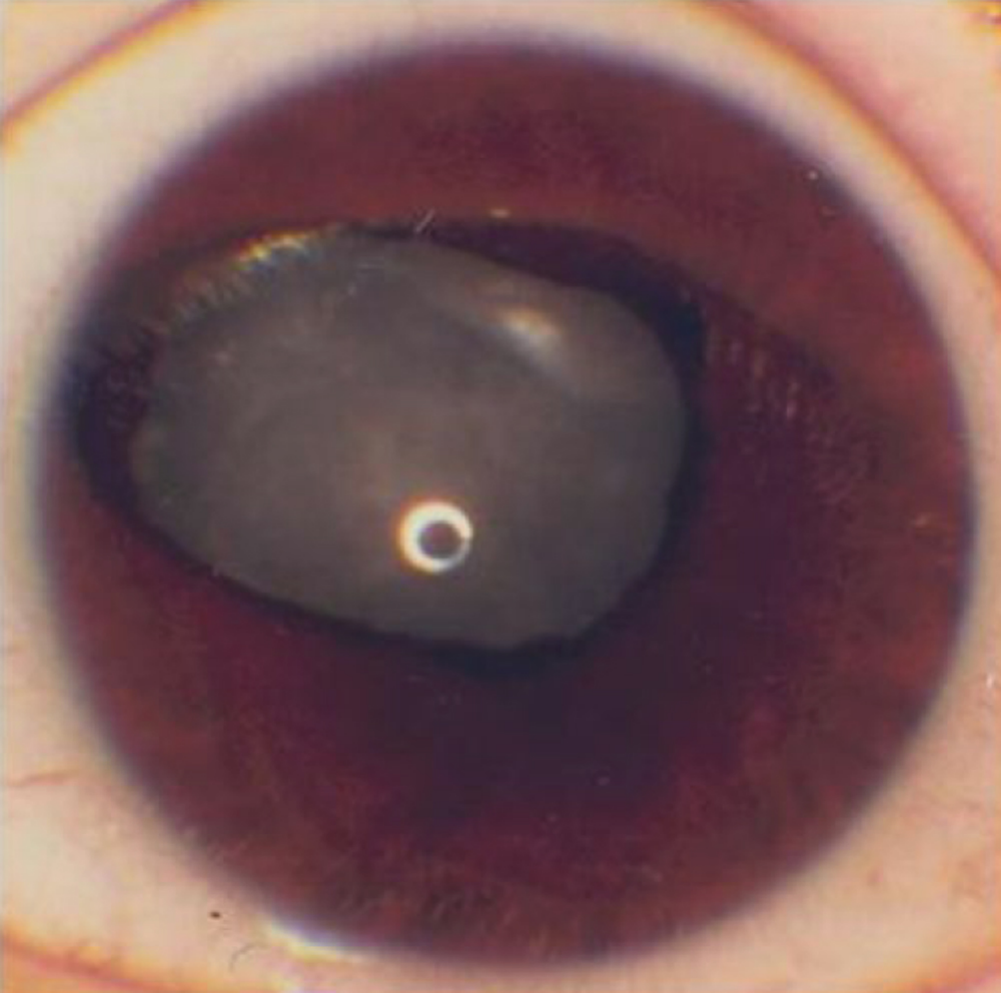
Left eye iris of aniridia case 6 who had a novel missense mutation (c.361T>C) located in the NH_2_-region of the paired domain of *PAX6*. This eye exhibited partial absence of iris, an atypical sector nasal iris coloboma (Iris 4), stromal hypoplasia, and a total cataract.

Case 6 was a female patient with a novel missense substitution. Her right eye exhibited an eccentric pupil, circumpupillary iris hypoplasia (Iris 3), and cortical cataract. In the left eye, she had an atypical sector nasal iris coloboma (Iris 4), stromal hypoplasia, and total cataract (Figure 1 and Figure 2). The missense mutation identified was c.361T>C in exon 7 that changes serine 121 to proline (p.S121P) in the paired domain. Her mother exhibited foveal hypoplasia and nystagmus with whole irides, and her sister had congenital cataracts, nystagmus, and macular hypoplasia. Both affected relatives had the mutated allele.

A base duplication at position 879 in exon 10 was found in case 21 and his mother. This previously unreported duplication (c.879dupC) causes a frameshift and introduces a premature stop codon 47 nucleotides downstream in the PST domain. The patient had Iris 5 with the associated ocular abnormalities of macular hypoplasia and nystagmus. The clinical manifestations of his mother were not available.

Female case 22 showed the novel missense substitution, c.277G>A, in exon 6, which encodes part of the extreme amino end of the paired domain. The mutation changes glutamate at position 93 to lysine. This case also had a previously reported intronic polymorphism (IVS9–12C>T) [21]. The patient presented with total aniridia (Iris 6), nystagmus, and congenital cataracts. Her mother was referred to as affected, but we could not accomplish family studies because the patient resided in an orphanage.

With respect to previously reported mutations, we found the IVS6+1G>C splice-site mutation [22] in case 18 who had Iris 5, microcornea, nystagmus, ectopia lentis, and macular and optic nerve hypoplasia. Her unaffected parents did not show this splice site change. Additionally, the patient and her father showed the previously described intronic substitution, IVS2+9G>A [20]. We searched for this substitution in 103 Mexican healthy controls and observed 19 heterozygotes (G/A) and two newborns homozygous for the A allele.

The only deletion that we observed was the previously reported loss of cytosine at position 853 (c.853delC) that introduces a premature stop codon 43 nucleotides downstream [5]. This deletion was found in case 20, but phenotypic information was not available.

We found the c.969C>T nonsense substitution (Human PAX6 allelic database), which changes arginine 203 to a UGA stop codon in the linker region, in two unrelated probands (case 10 and case 24); both were sporadic aniridia cases. Unfortunately, phenotypic information on case 24 and his parents were unavailable, but the molecular study was normal in both parents. Case 10 was a female dizygotic twin who showed subtotal aniridia (Iris 5), nystagmus, and macular hypoplasia. Her male twin and mother were genotypically normal and had a normal ocular phenotype, but the father was not studied.

Finally, we also observed a mutation that produces a substitution in the splice acceptor site of intron 7 (IVS7–2A>G). An in silico analysis of this mutation, which has been previously reported in another single study [22], revealed the possible use of different cryptic splice sites. The individual with this mutation (case 26) had Iris 6 with nystagmus, cataract, and strabismus. Other members of her family were referred to as having a normal ocular phenotype, but they were unavailable for study.

## Discussion

To the best of our knowledge, this is the first work on aniridia, apart from the original report, that uses the Gronskov classification of iris hypoplasia. Gronskov originally reported that the proportion of patients with Iris grade 1 to 4 was approximately 40% [5]  whereas we found only two index cases (7%), one with Iris grade 3 and 4, another with Iris 4, and none with lesser severity. This discrepancy might be explained by ascertainment bias, reflecting the fact that first-contact ophthalmologists are more familiar with the classic or severe aniridia presentation than with milder phenotypes. Another reason might be that individuals with milder cases, which are generally asymptomatic, do not seek medical care. In our opinion, Gronskov’s classification [5] should be widely used as a way to improve diagnosis, detect potential complications, and provide genetic counseling in aniridia cases with milder phenotypes.

To our knowledge, this work represents the third largest aniridia series (only smaller than those published by Gronskov et al. [14] and Vincent et al. [15]) that included a molecular study of *PAX6*. Although we analyzed the entire coding region of the *PAX6* gene in this work, the mutation detection rate of 30% that we found was lower than the 80% and 55% rates reported by the groups of Gronskov et al. [14] and Vincent et al. [15], respectively, who used diverse techniques for detecting pathological mutations. In this work, we used the SSCP technique exclusively, which is a widely used and efficient method for detecting mutations in *PAX6* [12,16]. However, a low rate of *PAX6* mutation detection (40%) using the SSCP technique has also been reported in patients described by Glaser et al. who proposed the possibility of mutations in more distant *cis* regulatory sequences [9]. Our low detection rate might be consistent with this interpretation because contiguous regulatory or non-coding sequences were not analyzed in our study. However, it also could be because of limitations of the SSCP technique itself as large genomic rearrangements would not be identified by this methodology. The inclusion of other mutation detection techniques in future studies would be expected to improve our mutation detection rate.

We identified eight different causal *PAX6* mutations in nine unrelated cases with isolated aniridia. The nature of the mutations was very similar to that reported in other populations [5,13,15,21]. Interestingly, we did not find the intragenic deletions previously reported in five Mexican patients, suggesting that these deletions might not be as frequent in our population as thought by Ramirez-Miranda et al. [17]. In this same context, our findings do not provide support for a founder effect of a specific mutation in the Mexican population [17].

The only intragenic deletion identified (c.853delC) produces a frameshift and introduces a premature stop signal 42 codons downstream in exon 8. If it were translated, the predicted truncated PAX6 product would retain the paired domain but lack the homeobox and PST transactivator domain. This mutation has been observed twice before, once in a male patient with aniridia (Iris 4), cataracts, and nystagmus [5] and once in a female in which only aniridia was mentioned [22]. Unfortunately, our case was unavailable for phenotype-genotype correlation.

The duplications, c.184_188dupGAGAC and c.879dupC, are novel, and both give rise to frameshifts, introducing premature stop codons in the paired domain and PST region, respectively. Phenotypes observed in other cases with insertion mutations are severe [5,23]. Consistent with this, our cases with these mutations had Iris 5.

The nonsense substitution, c.969C>T, which changes an arginine codon (CGA) to a stop codon (UGA), was detected in two unrelated, sporadic cases (cases 10 and 24). This mutation has been previously found in at least 20 patients worldwide including familial and sporadic cases, making it one of the three more frequent changes in *PAX6* along with c.1080C>T (27 cases) and c.1311C>T (20 cases; Human PAX6 allelic database). The differences in the ethnic origins of patients bearing the c.969C>T change indicate that this mutation is recurrent in *PAX6*. The recurrence of these three mutations might be explained at least in part by the presence of CpG dinucleotides in *PAX6* that tend to become methylated and might thereby create conditions favorable for C>T substitutions as a consequence of spontaneous deamination of cytosine residues [23]. Our two patients positive for c.969C>T might represent independent mutational events since they were unrelated.

With respect to the phenotype of c.969C>T heterozygotes, there are only five cases described in the Human PAX6 allelic database. Interestingly, one had partial aniridia with foveal hypoplasia and nystagmus, and the other four had aniridia with the associated ocular manifestations of nystagmus, cataracts, glaucoma, or corneal erosion. Of our two patients positive for c.969C>T, clinical information was available for only case 10. This patient had a severe phenotype and was classified as Iris 5 with nystagmus and macular hypoplasia.

Literature reports based on the haploinsufficiency model have suggested that frameshift and nonsense mutations predicted to result in a truncated protein such as those described above are likely to exert their pathological effects through a “nonsense-mediated-decay” process where translation to protein might not occur because the mRNA is degraded [21,23]. However, it has also been noted that truncating mutations located downstream of DNA-binding domains especially those in exons 12 and 13 might have a dominant-negative effect [23,24]. In the present work, we did not identify nonsense mutations in this extreme 3′ region of the *PAX6* gene.

On the other hand, both novel missense mutations observed in the present work–c.277G>A (p.E93K) and c.361T>C (p.S121P)–might affect the function of the paired-box domain of the PAX6 protein because the properties of the substituted amino acids are quite different. In one case (p.E93K), a negatively charged glutamate is replaced by a positively charged lysine. In the other (p.S121P), the polar serine residue is replaced by the non-polar amino acid, proline. Moreover, glutamate 93 and serine 121 are largely invariant among closely related PAX family members with glutamate 93 conserved in PAX3, PAX4, and PAX7 and serine 121 conserved in eight *PAX* family genes (Protein BLAST). An in silico analysis using the SIFT program predicted that protein function would be affected (p<0.01), providing support for a possible pathogenic effect of these mutations, but further functional analyses are needed to confirm this.

Missense mutations, which account for roughly 17% of changes in *PAX6* worldwide, potentially retain residual protein activity and have been associated with milder phenotypes [5,16,23]. Consistent with this, case 6 who had a c.361T>C mutation showed Iris 3 (circumpupillary iris hypoplasia) and Iris 4 (atypical sector coloboma), which were the mildest iris grades found in the probands of our series. In contrast, case 22 carrying a c.277G>A substitution had complete aniridia (Iris 6) as well as nystagmus and cataracts. Although both of these mutations affect the paired domain, the c.277G>A mutation is located in the NH_2_-region and would therefore be expected to have a more profound effect on paired domain structure and function than the COOH-terminally localized c.361T>C mutation. This difference in location may account for the observed phenotypic differences, but additional studies will be required to support this idea.

In some cases, missense mutations in *PAX6* have also been associated with neurodevelopmental abnormalities such as absence/hypoplasia of the anterior commissure, callosal area, or pineal gland; olfactory system anomalies; cerebellar coordination problems; mental retardation; and epilepsy [11,16,20,25-28]. In fact, Dansault et al. [20] suggested that these abnormalities should be systematically investigated in every patient with aniridia. In cases 6 (age 8 years) and 22 (age 15 years) who had the missense mutation, clinical neurological anomalies were not observed, but cerebral CT scan or MRI imaging were not performed. Further descriptions of aniridia cases with missense mutations and neurodevelopmental anomalies will be needed to improve genotype-phenotype correlations. In addition to the novel missense substitution, c.277G>A, female case 22 had the intronic polymorphism, IVS9–12C>T, which is thought to represent a neutral variant [21].

With respect to the splice-site mutation, IVS7–2A>G [22], an in silico analysis performed with the NetGene2 Server predicted that this change would eliminate the activity of the natural acceptor site in intron 7 and activate different cryptic acceptor sites within the exon or intron 8. It could, however, result in the use of the natural acceptor site in intron 8 and thereby lead to an in-frame, exon-skipping event that deletes exon 8. This mutation has been previously observed in a single case [22] with aniridia, cataracts, nystagmus, and corneal dystrophy (Human PAX6 allelic database). Similarly, our patient with this mutation (case 26) had a complete iris defect (Iris 6), nystagmus, cataract, and strabismus but without the corneal anomalies that might be present at an older age.

The previously reported IVS6+1G>C substitution [22] disrupts the conserved dinucleotide GT in the intron 6 splice-donor site and might lead to the use of an alternative in-frame donor site inside exon 6. The predicted protein would lack the last 36 amino acid residues encoded by this exon, and the resulting deletion of a portion of the paired domain would be expected to lead to a severe phenotype (Human PAX6 allelic database). Consistent with this, the ocular phenotype of our patient was Iris 5 with nystagmus, microcornea, ectopia lentis, and macular and optic nerve hypoplasia. Both parents were considered healthy and were negative for IVS6+1G>C. This mutation has been reported once before in an aniridia patient but without the description of other clinical data [22]. Remarkably, there have been at least nine previous reports of a substitution at guanine by either adenine or thymine in the +1 position in GT donor sites in aniridia patients [12,29,30].

In addition, case 18 and her unaffected father showed the previously described IVS2+9G>A substitution [20]. Although this intronic change was assumed to be potentially pathogenic by Dansault et al. [20] who observed it in a sporadic case with microphthalmia and other ocular abnormalities but not in 200 normal healthy individuals, an in silico analysis of this variant predicted that the binding capacity of the natural donor site would be unchanged. In our own search of 103 healthy Mexican newborns, we found this variant in a heterozygous state in 19 individuals and in a homozygous state in two. Hence, our data indicate that IVS2+9G>A is a neutral polymorphism and is not responsible for a pathological phenotype. The allele frequencies obtained for this polymorphism were in Hardy–Weinberg equilibrium.

In summary, most of the mutations detected in our analysis alter invariant amino acid residues in the paired domain or predict truncation of the PAX6 protein. Four of the *PAX6* mutations identified in this study are novel. In addition, our results lend support to the notion that c.969C>T is one of the three more frequent causal mutations in isolated aniridia cases and provide evidence that the IVS2+9G>A (c.-129+9G>A) variant is a neutral polymorphism.
